# Mindfulness-Based Smoking Cessation Delivered Through Telehealth and Text Messaging for Low-Income Smokers: Protocol for a Randomized Controlled Trial

**DOI:** 10.2196/35688

**Published:** 2022-08-01

**Authors:** Claire A Spears, Josephine Mhende, China Hawkins, Vuong Van Do, Matthew J Hayat, Michael P Eriksen, Donald Hedeker, Lorien C Abroms, David W Wetter

**Affiliations:** 1 Department of Health Policy & Behavioral Sciences School of Public Health Georgia State University Atlanta, GA United States; 2 Department of Population Health Sciences School of Public Health Georgia State University Atlanta, GA United States; 3 Department of Public Health Sciences University of Chicago Chicago, IL United States; 4 Department of Prevention and Community Health Milken Institute School of Public Health George Washington University Washington, DC United States; 5 Department of Population Health Sciences University of Utah and Huntsman Cancer Institute Salt Lake City, UT United States

**Keywords:** mobile health, mHealth, telehealth, SMS text messaging, mindfulness, smoking cessation, tobacco, health disparities, mobile phone

## Abstract

**Background:**

Tobacco use is the leading cause of preventable morbidity and mortality. Adults with low income and members of certain racial and ethnic minority groups are less likely to quit, and therefore, they experience profound tobacco-related health disparities. Mindfulness training can increase the rates of smoking cessation and lapse recovery, and telehealth and SMS text messaging have the potential to provide more accessible treatment.

**Objective:**

This study aims to test the efficacy of delivering mindfulness-based smoking cessation treatment through text messaging (iQuit Mindfully) and telehealth (group videoconferencing), both as stand-alone interventions and in combination. In addition, it aims to examine the underlying mechanisms of mindfulness treatment.

**Methods:**

In this 2×2 randomized controlled trial, participants are randomized into 1 of 4 groups based on assignment to iQuit Mindfully text messages (yes or no) and mindfulness videoconference groups (yes or no). The primary outcomes are biochemically verified smoking abstinence at 8, 12, and 24 weeks after the start of treatment. Secondary outcomes include the frequency of home mindfulness practice and self-reported levels of mindfulness, emotions, craving, withdrawal, dependence, self-efficacy, and social support.

**Results:**

Recruitment, treatment, and assessment began in spring and summer 2021, and data collection is expected to continue through spring 2024.

**Conclusions:**

This project aims to improve smoking cessation outcomes for low-income, racially and ethnically diverse smokers through mindfulness-based telehealth group counseling and text messaging support. We also aim to advance the scientific study of the mechanisms of action of mindfulness treatment, which could inform the development of more efficacious and efficient treatments to reduce tobacco disparities.

**Trial Registration:**

Clinicaltrials.gov NCT04965181; https://clinicaltrials.gov/ct2/show/NCT04965181

**International Registered Report Identifier (IRRID):**

PRR1-10.2196/35688

## Introduction

### Background

Tobacco use, the leading cause of preventable morbidity and mortality [1], disproportionately affects low-income and African American populations [2]. Both low-income and African American smokers have higher incidence and mortality rates of tobacco-related cancers [3-5]. Furthermore, cigarette smoking is more prevalent among adults living below the federal poverty line (25.3%) compared with those at or above the poverty level (14.3%) [6]. Smoking cessation greatly reduces the risk of cancer and related death [7,8], and most smokers in the United States (regardless of income, race, or ethnicity) want to quit smoking. However, only 7% quit each year [9], and the rates of successful smoking cessation are even lower among low-income and African American adults [2,9]. There is an urgent need to improve evidence-based smoking cessation interventions to better serve these populations.

Mindfulness training can increase the rates of smoking cessation and lapse recovery [10-12]. These programs teach individuals to observe present-moment sensations without judging or reacting to them. For example, mindfulness involves moment-to-moment awareness of stress, unpleasant emotions, and craving (all of which can be potent triggers for smoking) so that people can purposefully choose how to respond, rather than automatically react to the triggers by smoking [13]. Most research on mindfulness-based interventions (MBIs) has focused on higher-income and non-Latino White populations. However, MBIs have shown benefits for smoking cessation in low-income [14] and racially and ethnically diverse adults [10,11]. In addition, mindfulness-based relapse prevention has been reported to be more efficacious than traditional relapse prevention in reducing drug use among racial and ethnic minority women [15]. In a sample of racially and ethnically diverse adult smokers, higher mindfulness was indirectly associated with a lower likelihood of smoking lapse through its association with reduced stress [16]. Moreover, among low-income African American adults, the ability to practice mindfulness on one’s own, without having to rely on external resources, has been described as helpful for managing stress and improving health [17].

Although mindfulness practice does not require substantial resources, the mindfulness interventions that have undergone the most empirical testing involve considerable time and resources. Mindfulness-based stress reduction (MBSR) [18] and mindfulness-based cognitive therapy (MBCT) [19] are typically provided by highly trained instructors in 8 weekly, in-person 2- to 2.5-hour sessions, plus a day-long retreat. Although these programs are beneficial for those with the requisite time, transportation, and financial and other resources, there is a need for more cost-effective and scalable MBIs for a broader reach. For example, offering MBIs through telehealth (eg, group videoconferencing) could improve treatment access. A 2020 systematic review supported the feasibility and acceptability of delivering MBSR and MBCT via group videoconferencing, with moderate positive effects on mental health outcomes compared with inactive controls and no significant differences compared with in-person delivery [20]. A broader 2021 systematic review of web-based MBIs (including MBIs other than MBSR and MBCT as well as various forms of guided and unguided web-based delivery) found pre-post intervention benefits for well-being, depression, anxiety, stress, and mindfulness [21]. Therapist-guided web-based MBIs were superior for reducing stress compared with unguided web-based MBIs [21]. To our knowledge, mindfulness-based smoking cessation treatment has not been delivered through group videoconferencing, although MBIs for smoking cessation have been provided through web-based prerecorded video instructions with telephone counseling support [22] and smartphone apps [23,24]. Given the safety concerns with in-person groups during the COVID-19 pandemic and our goal of developing more scalable interventions, we recently transitioned mindfulness-based addiction treatment (MBAT), an 8-week group-based smoking cessation intervention, from in-person to web-based delivery [25]. The first 5 weekly sessions were conducted in person, with the last 3 successfully delivered through group videoconferencing. Providing all 8 MBAT sessions on the web could further improve access to treatment.

In addition, innovative methods are needed to increase MBI engagement and promote adherence to mindfulness practice in daily life; that is, MBIs encourage participants to practice mindfulness between sessions, and consistent home practice is hypothesized to be a key factor in producing therapeutic outcomes [26]. However, mastering a new skill in treatment does not necessarily mean that it will translate to real-life situations, and a consistent barrier to treatment success occurs when individuals fail to practice mindfulness in daily life. MBI participants often do not practice mindfulness as frequently as directed [11,26]. Relatedly, the lack of support between weekly MBI sessions could limit the effectiveness of the intervention. Additional support may be needed for low-income smokers who experience significantly more stress and smoking-conducive contexts on a day-to-day basis [16,27] and have lower health care access [2]. Mobile health (mHealth) technology (eg, delivering interventions through text messaging) offers promise to (1) deliver more cost-effective and scalable treatment, (2) increase engagement with mindfulness home practice, and (3) provide vital 24/7 support to improve smoking cessation outcomes for low-income smokers. For example, text messages could encourage people to use mindfulness coping strategies amid day-to-day stress, cravings, and other challenges. As text messaging does not require a smartphone, internet access, or advanced technical skills, this intervention modality could be appropriate for individuals with lower socioeconomic resources. Indeed, there is strong empirical support for text messaging interventions for smoking cessation [28].

On the basis of iterative feedback from low-income smokers, we developed a text messaging program (*iQuit Mindfully*) as an adjunct to in-person, mindfulness-based smoking cessation treatment [29,30]. In a pilot study (N=71), participants were highly engaged and benefited from tailored, in-the-moment strategies and social support from text messages [29]. Notably, although poverty status predicted worse cessation outcomes among participants receiving only in-person treatment, poverty status was unrelated to cessation among those receiving iQuit Mindfully. In fact, 23.1% of participants living in poverty who received iQuit Mindfully achieved biochemically confirmed abstinence at the end of treatment and at the 1-month follow-up, whereas none of those living in poverty quit in the in-person-only treatment. Building on this initial work, we incorporated participant feedback to further increase interactivity and personalization so that iQuit Mindfully can more flexibly adapt to the changing needs of the participants. We also developed a version of iQuit Mindfully that can be implemented as a stand-alone program (ie, without weekly mindfulness-based group sessions) [25].

In addition to delivering MBIs via telehealth (eg, group videoconferencing) and mHealth (eg, text messaging), mHealth methods could further our scientific understanding of how treatments work. Researchers have highlighted the need to clarify the mechanisms of action of MBIs [31-33]. Intensive longitudinal assessment on mobile phones could elucidate how mindfulness impacts smoking behavior on a day-to-day basis, which could inform the development of more efficacious and efficient treatments. Our conceptual framework, rooted in social cognitive theory [34], relapse prevention theory [35], and past work on MBI mechanisms [13,32,36], describes the hypothesized mechanisms by which MBIs enhance smoking cessation outcomes. First, MBIs are thought to increase dispositional mindfulness (ie, present-focused, nonjudgmental attention in everyday life), which predicts better smoking cessation outcomes [37,38]. Second, mindfulness appears to impact emotions and emotion regulation, which are critical to quit smoking. Escaping, avoiding, or reducing negative affect are key drivers of substance use [39]. In the “addictive loop” [13], unpleasant cues elicit negative affect, which triggers craving and smoking. MBIs have been shown to reduce negative affect [40] and its volatility (ie, lability or instability) [36]. Although more mindfulness research has studied the effects of negative than positive emotions, mindfulness might also increase positive affect [41], which could be protective in the process of quitting [42]. Third, mindfulness may reduce craving and withdrawal [36]. That is, nonjudgmental observation of craving could diminish the intensity of unpleasant sensations associated with craving and withdrawal.

Fourth, mindfulness might weaken associations of both negative affect and craving with smoking, thus targeting key aspects of the addictive loop [13]. Even when smokers inevitably experience craving and negative affect, mindfulness moderates their responses to these experiences. For example, “urge surfing” encourages smokers to nonjudgmentally observe cravings as ocean waves that rise and eventually pass [43]. People learn that cravings are transient sensations that they can “ride out” without smoking. Previous research suggests that mindfulness “decouples” the links among negative affect, craving, and substance use [13,44,45]. Fifth, mindfulness may increase self-efficacy for abstaining from smoking in high-risk situations [36]. Low self-efficacy often predicts the lapse and relapse of smoking [46]. By teaching nonreactive observation of smoking triggers, MBIs might increase confidence in one’s ability to encounter triggers without smoking. Finally, providing mindfulness treatment through videoconferencing groups and text messaging could enhance the perceived social support of the participants, which predicts better cessation [47]. Participants in our pilot study with iQuit Mindfully indicated feeling a sense of social support and accountability from the text messages [29,30].

### Objectives

This randomized controlled trial aims to (1) test the efficacy of delivering mindfulness-based smoking cessation treatment through text messaging (iQuit Mindfully) and telehealth (MBAT through group videoconferencing), both as stand-alone interventions and in combination, and (2) examine the underlying mechanisms of mindfulness treatment. On the basis of clinical trial results, iQuit Mindfully and videoconference-delivered mindfulness treatment for smoking cessation could be highly scalable and cost-effective.

## Methods

### Study Design, Aims, and Hypotheses

This study is a 2×2 randomized controlled trial to investigate the effects of the iQuit Mindfully text messaging program and telehealth-delivered MBAT, both as stand-alone interventions and in combination. Approximately 485 participants will be randomized into 1 of 4 groups based on assignment to iQuit Mindfully text messages (yes or no) and MBAT videoconference groups (yes or no). The four conditions are Usual Care (self-help materials and nicotine replacement therapy [NRT]), MBAT (8 weekly videoconference group MBAT sessions, self-help materials, and NRT), iQuit Mindfully (iQuit Mindfully text messages, self-help materials, and NRT), and MBAT+iQuit Mindfully (8 weekly videoconference group MBAT sessions, iQuit Mindfully text messages, self-help materials, and NRT). Assessments are performed at baseline and weeks 1, 3, 5, 8, 12, and 24.

The primary outcomes are smoking abstinence at 8 weeks after the start of treatment (7-day abstinence, biochemically verified by expired carbon monoxide [CO] <6 ppm), 12 weeks after the start of treatment (3-month follow-up; 7-day abstinence, verified by CO <6 ppm), and 24 weeks after the start of treatment (6-month follow-up; 7-day abstinence, biochemically verified by saliva cotinine <20 ng/mL). Secondary outcomes include the frequency of home mindfulness practice and self-reported levels of mindfulness, emotions, craving, withdrawal, dependence, self-efficacy, and social support.

The aims and hypotheses are subsequently outlined.

The first aim is to test the efficacy of a mindfulness-based text messaging program for smoking cessation (iQuit Mindfully) and telehealth-delivered MBAT, both as stand-alone interventions and in combination.

We hypothesize the following:

- MBAT+iQuit Mindfully will result in higher rates of smoking cessation and lapse recovery than MBAT or iQuit Mindfully alone. MBAT and iQuit Mindfully, as stand-alone interventions, will also produce higher cessation and lapse recovery than usual care.
- As an exploratory aim, we will examine whether poverty status moderates treatment efficacy (ie, poverty status will predict worse cessation outcomes in MBAT but not in MBAT+iQuit Mindfully, which provides 24/7 mHealth support).

The second aim is to investigate the mechanisms through which mindfulness training impacts smoking cessation. Participants will complete questionnaires from baseline to 24 weeks after the start of treatment, in addition to intensive diary assessments for 6 contiguous weeks during treatment. We hypothesize the following:

- Compared with usual care, the mindfulness treatment arms (MBAT, iQuit Mindfully, and MBAT+iQuit Mindfully) will increase mindfulness, reduce negative affect and volatility of negative affect (ie, greater affective stability), increase positive affect, reduce craving and withdrawal, and increase self-efficacy and social support, all of which will mediate the effects of mindfulness-based treatment arms versus usual care on abstinence.
- Compared with usual care, MBAT, iQuit Mindfully, and MBAT+iQuit Mindfully will all attenuate the links between links between negative affect and craving with smoking. That is, in addition to reducing negative affect and craving, mindfulness training is hypothesized to weaken the relationships between negative affect and craving with smoking.
- Compared with MBAT, MBAT+iQuit Mindfully will produce stronger effects on the mechanisms mentioned above. The mechanisms outlined in the mindfulness treatment arms will mediate the effects of MBAT+iQuit Mindfully versus MBAT on abstinence.

### Ethics Approval and Registration

This study was approved by the Georgia State University (GSU) institutional review board (protocol H20479) and was registered on clinicaltrials.gov (NCT04965181).

### Participants and Recruitment

The following are the inclusion criteria: a minimum age of 18 years; current smoker with a history of >3 cigarettes/day (and expired CO >6 ppm); motivation to quit smoking within the next 30 days; a valid home address in the greater Atlanta, Georgia, area; a functioning telephone number; and the ability to speak, read, and write in English. The following are the exclusion criteria: contraindication for nicotine patch or nicotine lozenge; current problematic substance use or clinically significant depressive symptoms; current use of tobacco cessation medications; pregnancy, planning to become pregnant in the next 5 months, or lactation; household member enrolled in the study; or those enrolled in previous studies on iQuit Mindfully at GSU.

Recruitment focuses on flyers; print, radio, and social media; community outreach; and partnerships with local health care systems, with targeted recruitment of low-income and racially and ethnically diverse smokers in Atlanta, Georgia. Flyers are posted at and near local clinics and community health centers, community organizations, and shelters; on and near GSU campuses; and near local train and bus stops. The research team also attends meetings sponsored by county and state health departments to communicate with stakeholders and hand out recruitment flyers. Web-based recruitment strategies include advertising posted on the Craigslist and Nextdoor websites.

### Study Interventions

#### Nicotine Replacement Therapy (All Conditions)

Participants are provided with 8 weeks of nicotine patches and nicotine lozenges, with dosages depending on the number of cigarettes per day and time to first cigarette upon waking. Participants receive 4 weeks of nicotine patches and lozenges at baseline, and they are mailed the additional 4 weeks of NRT when needed.

#### Self-help Materials (All Conditions)

All participants are given evidence-based self-help materials for smoking cessation (based on the Treating Tobacco Use and Dependence Clinical Practice Guideline) [48]. Materials include the “Clearing the Air” booklet published by the National Cancer Institute and a referral to the Tobacco Cessation Quitline (1-800-QUIT-NOW).

#### MBAT (MBAT and MBAT+iQuit Mindfully Conditions)

Participants in the MBAT and MBAT+iQuit Mindfully conditions receive videoconference group counseling (using the Zoom platform) based on the MBAT manual (D Wetter, unpublished data, June 2009). Instructors are certified MBSR teachers and licensed clinicians who received additional training on tobacco cessation. MBAT consists of 8 weekly 2-hour sessions. Participants are encouraged to choose their own quit date between day 7 and day 30 of the program. MBAT sessions teach participants to notice the tendency for mindlessness (“automatic pilot”) and focus attention on the present moment, including observing sensations of craving and difficult emotions, so that they can choose to cope in adaptive ways other than smoking. MBAT emphasizes daily mindfulness practice in several forms including sitting meditation, body scan meditation, walking meditation, eating meditation, and gentle yoga. The program also teaches cognitive behavioral strategies for smoking cessation, including clearing the environment of smoking cues, recognizing and coping with high-risk situations, and managing stress [11].

Several strategies are being implemented to promote engagement with telehealth-delivered MBAT among adults of low-income populations. For example, participants in the MBAT and MBAT+iQuit conditions can borrow a study tablet for the 8 weeks of the program. Participants are also given the option to join videoconference groups in an individual room at our research office, in cases in which they do not have a private place to join groups. Finally, the research team provides guidance and technical support for using Zoom through an in-person baseline session, Zoom group orientation session, and ongoing troubleshooting as needed.

#### iQuit Mindfully Text Messages (iQuit Mindfully and MBAT+iQuit Mindfully Conditions)

Participants receive text messages throughout the 8-week treatment period as well as less frequent messages during the follow-up period, depending on their preferences. All messages are automated using the Upland Mobile Commons platform. Participants are asked to set a quit date at baseline, which is integrated into text messages and can be changed by the participant. Text messages encourage participants to practice mindfulness and other strategies for smoking cessation (for more details, refer to the study by Spears et al [29]). The messages are designed to be interactive. That is, participants are asked questions through a series of flow logic (eg, “Would you like to practice mindfulness right now?” If participants replies “yes,” they are provided a mindfulness technique, later asked about how it went, and are encouraged to continue practice). Participants can also text CRAVE, STRESS, SLIP, or FACT at any point to receive additional text message support for coping with cravings, stress, smoking lapses, or to receive facts about the effects of smoking, respectively. Participants can answer “group poll” questions so that they later receive a text with the most common (deidentified) responses. Text messages include quotes sharing the anonymous experiences of former smokers in our program. Participants can also text keywords (MIND, BODY, or 3MIN) to receive a phone call with a short recording of a mindfulness practice. Participants are given small pocket cards with basic information on the text messaging program and reminders about the text keywords.

Text messages are personalized based on first names, personal reasons for quitting, and the amount of money to be saved based on individual smoking habits and price paid per pack. Picture messages are included based on our initial qualitative work [30]. On the basis of the feedback from our previous message testing [29], message timing and frequency are flexible and personalized. Participants choose the frequency of their choice (ie, very low to very high, ranging from 1 to 2 to 5 to 6/day) as well as a 12-hour time slot of their choice (7 AM to 7 PM or 10 AM to 10 PM). Text messages periodically ask participants about their preferred text message frequency and timing so that participants can change their message schedule as needed.

### Study Procedures

#### Overview

Individuals who are interested in the study either call the research office to complete telephone screening or click on a link to take a web-based screener survey. Eligible individuals are then scheduled for an in-person session to finalize eligibility, engage in further discussion about the study, and provide written informed consent. To confirm eligibility, the participants provide a breath sample for the assessment of expired CO. Individuals who decline or are ineligible are given self-help materials and referred to other cessation programs. In addition, potential participants complete the Patient Health Questionnaire-2 [49] and Severity of Dependence Scale [50] to screen for clinically significant depressive symptoms and substance dependence, respectively. Individuals who are ineligible for these reasons are provided with appropriate mental health referrals in addition to smoking cessation referrals.

Eligible individuals who provide written informed consent are then asked to complete baseline questionnaires, followed by randomization. Permutated block randomization is implemented, with stratification by race and poverty status. Participants are provided with information and materials specific to their intervention condition and scheduled for their next study visits. Web-based surveys are administered at weeks 1, 3, and 5. In addition, electronic diary assessments (brief surveys sent via text message or email depending on participant preference) are administered in the evening on every other day from week 2 to week 8, thus capturing key processes surrounding quitting smoking. Diary assessments assess smoking behavior (“How many cigarettes did you smoke today?”) and each of the hypothesized mechanisms (mindfulness, emotions, craving and withdrawal, weakened associations of negative affect and craving with smoking, self-efficacy, and social support). In-person assessments take place at weeks 8, 12, and 24, including assessment of expired CO (all in-person visits) and salivary cotinine (week 24) for biochemical confirmation of smoking behavior. The descriptions of the questionnaires are provided below.

#### Smoking Behavior

Tobacco History assesses the onset of regular smoking, previous quit attempts, abstinence history, smoking rate, and partner smoking status. This includes the Heaviness of Smoking Index (HSI), which comprises two items from the Fagerström Test for Nicotine Dependence [51]: self-reported average number of cigarettes smoked per day and time to first cigarette upon waking (“time to first cigarette”). The HSI is a strong indicator of nicotine dependence [52]. Smoking status surveys tobacco use, use of other tobacco products, and nicotine replacement medications. Smoking abstinence is assessed according to the Society for Research on Nicotine and Tobacco guidelines [53]. The use of novel tobacco products (eg, e-cigarettes) is also assessed, and participants indicate which forms of NRT and/or pharmacotherapy (if any) they used in the past week. Additional resources used for smoking cessation assesses the use of various strategies for quitting smoking (eg, acupuncture, hypnosis, other text messaging programs, or mobile apps) outside of the treatment provided by the study.

The Brief Wisconsin Dependence Motives Questionnaire [54] is a 37-item measure that yields an overall dependence score and subscale scores for other dimensions (ie, cognitive enhancement, affective enhancement, automaticity, affiliative attachment, loss of control, craving, cue exposure or associative processes, social or environmental goals, taste or sensory processes, weight control, and tolerance). The Wisconsin Smoking Withdrawal Scale [55] includes subscales for anger, anxiety, sadness, concentration difficulty, craving, hunger, and sleep. The Self-Efficacy Scale assesses confidence in resisting smoking urges in specific situations (eg, when feeling stressed or when with friends) [56]. Subscales include negative affect, pleasure, social image, social influence, and diet.

#### Mindfulness

The Mindful Attention Awareness Scale [57] is a 15-item self-report measure of dispositional mindfulness [57]. The Five-Facet Mindfulness Questionnaire-Short Form [58] is a 24-item self-report questionnaire on facets of dispositional mindfulness (nonreactivity, observing, acting with awareness, describing or labeling with words, and nonjudging of experience). Participants also complete a Mindfulness Practice Log [11] to indicate how frequently they practice each mindfulness technique taught in treatment. The Self-Compassion Scale-Short Form [59] is a 12-item self-report measure of self-compassion. Subscales include self-kindness, self-judgment, common humanity, isolation, mindfulness, and overidentification.

#### Stress and Emotions

The Perceived Stress Scale [60] is a 10-item self-report measure of the extent to which individuals view their lives as stressful. The Positive and Negative Affect Schedule [61] is a 20-item self-report measure of affective experience, yielding two factors (positive and negative affect).

#### Social Support

The Multidimensional Scale of Perceived Social Support [62] is a 12-item scale, with subscales assessing perceived social support from family, friends, and significant others. The Group Climate Questionnaire [63] is a 12-item self-report scale that measures the climate and cohesion of the group (only applicable for MBAT participants).

#### Program Evaluation

Participants provide feedback about their experiences receiving the text messages (text message feedback). They also complete 4 items to indicate perceived benefits of the smoking cessation program. As described by Hoeppner et al [64], participants rate the extent to which the program gave them confidence to quit smoking, made them think it was worthwhile to quit, made them feel that someone cared if they quit, and made them feel that they knew the right steps to take to quit. Items are rated from 1 (completely disagree) to 5 (completely agree). Participants also complete program evaluations to provide feedback and suggestions for improving MBAT and iQuit Mindfully. Usual care participants answer one program evaluation question: “On the scale below, please circle the number that best represents whether you would recommend this quit smoking program (or something similar) for others who are interested in quitting smoking” (rated on a 1 to 10 scale).

#### Financial Compensation and Retention Procedures

Participants receive financial compensation for the time and inconvenience associated with participation as well as parking validation or train or bus vouchers to defray the cost of travel. Participants are compensated up to US $30 for web-based surveys at weeks 1, 3, and 5 (US $25 for each survey plus US $5 bonus if completed within 24 hours). They are also compensated US $40 for week 8, US $60 for week 12, and US $70 for week 24 (all in-person visits). In addition, participants are compensated US $5 per each of the every-other-day surveys (maximum US $120 for all 6 weeks). The maximum compensation per person for completing all aspects of the study is US $380.

Other procedures to increase adherence include the following: (1) reminder phone calls, e-mails, and text messages; (2) requiring a phone number and valid home address so that participants can be contacted; (3) loaning participants a mobile phone for the study duration if they do not have one; and (4) obtaining names, addresses, and phone numbers of up to 3 collaterals who can provide information on participants’ whereabouts if necessary. In addition to traditional procedures for the informed consent process, research staff members engage eligible individuals in a discussion of the pros and cons of participating as well as participants’ ambivalence about behavior change and research participation. These methods have been suggested to improve retention [65].

### Analytic Plan

#### Overview

We will conduct both intent-to-treat (ITT) and per-protocol analyses. ITT includes all randomized participants, regardless of compliance, withdrawal, and other events after randomization. A strength of ITT is that it is based on the original randomization. However, effect estimation using ITT may be conservative and misleading with increasing attrition. Per-protocol analysis considers only participants who fully complied and completed the study. Per-protocol analysis is less conservative and may reflect true treatment differences for patients with full compliance. Including both approaches will provide a more complete understanding of the treatment effects.

Analyses will be performed using the SAS software. All analyses will control for demographics (age, gender, race, education level, and partner status) and baseline nicotine dependence (cigarettes per day and time to first cigarette).

#### Aim 1: Intervention Efficacy

The relationship between the treatment condition and each dichotomous study outcome (smoking cessation and lapse recovery) will be assessed using a generalized linear mixed model with a binomial distribution and logit link function. This is an appropriate statistical model with repeated measures data collected over time [66]. A 2-level multilevel generalized linear mixed model will be specified, with time nested within the subject. This approach will properly account for the multilevel data structure. The fixed effects considered in each model will include demographics, baseline nicotine dependence, treatment condition and time as main effects, and a treatment condition by time interaction. Testing of the covariance structure will be conducted, and information criteria will be used to assess model fit and selection. Linear contrasts will be built into the analysis to estimate the specific comparative effects of all treatment conditions. To test treatment effects on primary smoking cessation outcomes, repeated measures outcomes will be biochemically confirmed at 7-day point prevalence abstinence at weeks 8, 12, and 24. To test the effects of treatment on lapse recovery, this analysis will also be conducted among participants classified as smoking at the last treatment session [11]. In addition, gender, race and ethnicity will be assessed as potential moderators of the treatment effects.

The moderating effect of poverty status will be examined with the addition of a poverty status by treatment condition interaction into the models described above. This will be tested using both dichotomous (ie, below vs at or above the federal poverty threshold) and continuous poverty status variables (ie, depth of poverty as indicated by the ratio of income to poverty and income deficit or surplus) according to the United States Census Guidelines [67,68].

#### Aim 2: Underlying Mechanisms

Hypotheses regarding the mechanisms will be tested separately using both traditional questionnaire data (collected from baseline to 24 weeks after the start of treatment) and electronic diary data (collected for 6 continuous weeks during treatment). Mediation testing will be conducted regardless of the statistical significance of the intervention effects because of statistical, conceptual, and practical reasons for testing mediation in such cases [69]. Volatility will be calculated using the mean square successive difference approach [70] to capture both within-person variability and temporal instability. Mediation will be examined using multilevel mediation analyses for binary outcomes in the developed generalized linear mixed model. Bootstrapping with 5000 replications will be used to estimate the standard errors and *P* values for indirect effects. In addition to the mediators of associations between treatment and abstinence outcomes, we will test more fine-grained day-to-day associations using electronic diary assessments. We will test moderated mediation (ie, the hypothesis that mindfulness not only reduces stress, negative affect, and craving but also weakens the associations between these variables and smoking) using a model proposed by Preacher et al [71], as the independent variable (mindfulness training) also functions as a moderator. Kline [72] termed this “second-stage moderation,” as the second path of the indirect effect of the predictor on the dependent variable depends on the moderator. Conditional mediation and moderation effects will be tested using bootstrapping methods [71].

#### Missing Data

Although there is no complete, comprehensive method for handling missing data for generalized linear mixed models, several different approaches can be used to address this issue [66]. We will use multiple imputations of missing data whereby missing observations for individuals are estimated based on baseline responses and other study covariates, and we will perform additional sensitivity analyses regarding various missing data assumptions for smoking outcomes [73].

#### Power and Sample Size Determination

The sample size was estimated based on the number of participants required to detect meaningful treatment effects of the iQuit Mindfully and MBAT interventions. We obtained effect sizes (converted to Cohen *d* for comparability across measures) from relevant meta-analyses and used the Optimal Design program to estimate the sample size for the generalized linear mixed models [74]. Smoking abstinence in MBIs versus usual care was quantified as Cohen *d*=0.35 in a meta-analysis [12], and a Cochrane review [75] of primarily text messaging–based interventions for smoking cessation concluded an effect size of Cohen *d*=0.33. Assuming 80% power, a level of significance of .05, 2-tailed statistical tests, an error variance of 1, a coefficient variance of 2, and oversampling to account for 20% attrition, this results in 145 participants in each treatment group. An unequal allocation ratio was used to allocate fewer participants to usual care (n=50) [76,77] for an overall sample size of 485 participants (145 per treatment group and 50 in usual care). We expect this sample size to adequately allow for mediational testing (aim 2) at the 80% power level. Fritz and MacKinnon [78] offer a guide regarding the sample size required to detect mediated effects and report that 79.9% of studies in their literature review applied mediational testing with <400 participants, suggesting that our study plan with 485 participants will be ample to achieve all study aims.

## Results

Recruitment, treatment, and assessment began in spring and summer 2021, and data collection is expected to continue through spring 2024. Participants are enrolled into sequential cohorts to allow participants in a given cohort to start the 8-week videoconference MBAT groups together. The final cohort is expected to complete treatment by the end of 2023 and the final 24-week assessments in the first quarter of 2024.

## Discussion

### Overview and Purpose of Study

This paper describes the design of a randomized controlled trial of telehealth and text messaging delivery for mindfulness-based smoking cessation treatment. This project aims to improve smoking cessation outcomes for low-income, racially and ethnically diverse smokers through mindfulness-based telehealth group counseling and text messaging support, both separately and in combination. An overarching goal is to increase the reach and accessibility of mindfulness for more diverse populations, given that most mindfulness research has focused on higher-income and non-Latino White participants. We hypothesize that in this racially and ethnically diverse and predominantly low-income sample, the combination of mindfulness-based treatment through telehealth plus text messaging (MBAT+iQuit Mindfully) will result in higher rates of smoking cessation and lapse recovery than either program alone and that MBAT and iQuit Mindfully as stand-alone interventions will be superior to usual care. Furthermore, we aim to advance the scientific study of the mechanisms underlying mindfulness-based smoking cessation, which could inform the development of more efficacious and efficient treatments to reduce tobacco-related health disparities.

### Strengths, Limitations, and Future Directions

This study is strengthened by a rigorous experimental design to test the effects of mindfulness-based telehealth group counseling and text messaging, both as stand-alone interventions and in combination. The usual care control condition, including 8 weeks of combination NRT, self-help materials, and referral to the Tobacco Quitline, is a more robust comparison treatment than what participants would likely receive within standard health care. Intensive longitudinal data will be used to evaluate the underlying treatment mechanisms, which could inform the optimization of future treatments. The study is limited by the lack of follow-up after 6 months. Depending on the results of this trial, future trials might evaluate longer-term intervention effects (eg, 12- and 24-month follow-ups) and best practices for dissemination and implementation. It will be critical to ensure that effective treatments are accessible to low-income populations, for example, through partnerships with federally qualified health centers, community centers, the Tobacco Quitline, and/or other accessible resources. Future work might also investigate whether individual differences moderate treatment effects. For example, it is possible that participants with different sociodemographic or clinical profiles may benefit more from mindfulness, mHealth, or other treatment modalities. This information would be useful for practitioners to guide patients to treatment approaches best suited to their needs.

### Dissemination Plan

Findings will be presented at scientific meetings and published in a timely fashion, and the published manuscripts will be submitted to the digital archive PubMed Central. The research team will also work with our community collaborators to identify appropriate avenues to communicate project findings to community members in a way that is familiar, comprehensible, and relevant. The research team has formed connections with health care providers and facilities that serve underserved communities and will work to communicate findings through these organizations.

## Abbreviations

CO: carbon monoxide
GSU: Georgia State University
HSI: Heaviness of Smoking Index
ITT: intent-to-treat
MBAT: mindfulness-based addiction treatment
MBCT: mindfulness-based cognitive therapy
MBI: mindfulness-based intervention
MBSR: mindfulness-based stress reduction
mHealth: mobile health
NRT: nicotine replacement therapy
